# Supplementary material for: From deterministic to fuzzy decision-making in artificial cells
Source: Nat Commun. 2020 Nov 6;11:5648. doi: 10.1038/s41467-020-19395-4 (PMC7648101; doi:10.1038/s41467-020-19395-4)
Supplement: Supplementary file 1 — Supplementary Information [file 41467_2020_19395_MOESM1_ESM.pdf]

Supplementary Information for

From deterministic to fuzzy decision-making in artificial cells

Ferdinand Greiss[1], Shirley S. Daube[1], Vincent Noireaux[2], Roy Bar-Ziv[1,*]

[1]Department of Chemical and Biological Physics, Weizmann Institute of Science, Rehovot 76100, Israel

[2]Department of Physics, University of Minnesota, Minneapolis, Minnesota 55455, USA

*Corresponding author. e-mail: roy.bar-ziv@weizmann.ac.il

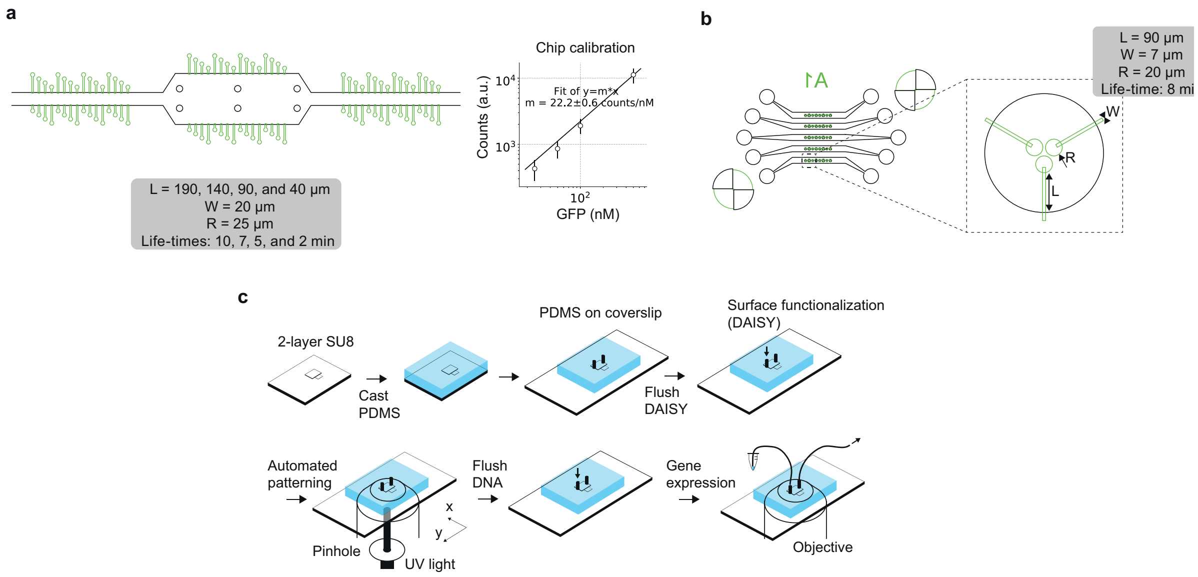

Supplementary Figure 1: Design and assembly of microfluidics chips.

a, Chip design for high molecule numbers in the high-density regime. The GFP concentration was calibrated by flushing known concentrations of purified GFP in 1x PBS. **b**, Chip design for low-gene density experiments on TIRF microscope. 5 lanes to perform multiple experiments at different conditions. **c**, PDMS chips are fabricated with 2-layer SU8 structures, flushing of chemicals directly onto the chip, and *in situ* DNA patterning and processing until gene expression is performed with *E. coli* cell extract (Methods).

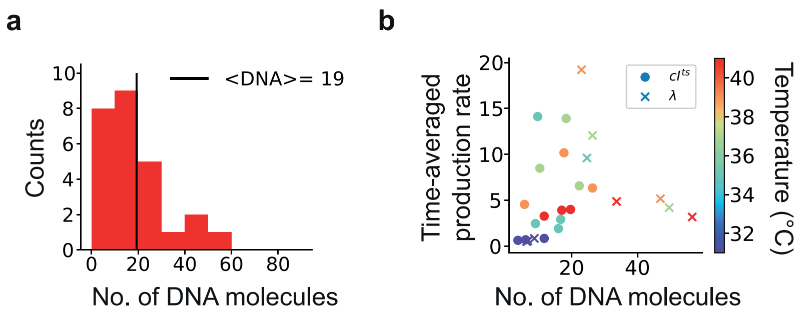

Supplementary Figure 2: Numbers of genes in compartments and time-average production rates during experiments at the low-density regime.

a, Histogram of detected DNA spots in individual compartments. **b**, Time-averaged production rates against the number of DNA spots (with bistable "λ" and monostable "cI^{ts}" GRN) in individual compartments at various temperatures (color-coded).

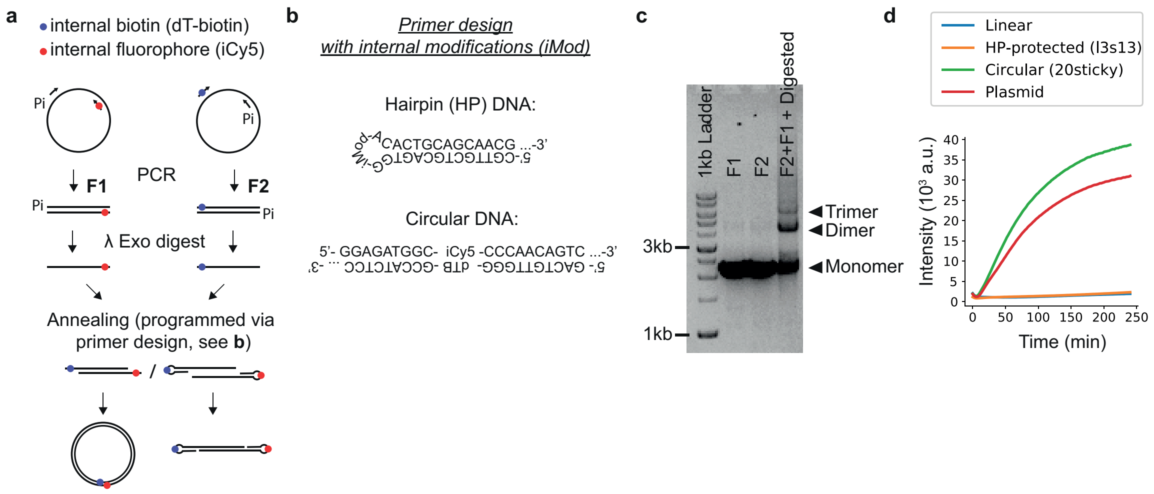

Supplementary Figure 3: DNA protection by circularization in *E. coli* extract.

a, 2-step protocol to modify the ends of double-stranded DNA fragments with PCR. Step 1: Two separate PCR reactions are started with two primer pairs (Methods, Supplementary Table 1) on the same plasmid. Step 2: Digestion of double-stranded PCR fragments gave single-stranded DNA fragments. The selective digestion of one strand by lambda exonuclease was controlled with phosphorylated primers. The two single-stranded DNA fragments annealed simultaneously to give a double-stranded fragment with end modifications, e.g. circular DNA or hairpin structure. **b,** Annealed primers shown to program for circular and hairpin products. **c,** Agarose gel with ladder (1kb DNA ladder, ThermoFisher Scientific), F1, F2, and digested F1+F2 fragments (from left to right). The circular DNA could be identified after annealing F1 and F2. Higher bands (dimers and trimers) are formed through self-binding at high concentrations. A control gel was run for each preparation (N>3). **d,** GFP production in solution experiments from different DNA sources ("Linear" = linear dsDNA as negative control; "HP-protected (l3s13)" = Hairpin structure with 3 and 13 bases in loop and stem region, respectively; "Circular (20sticky)" = circular DNA with 19 (=20 - 1 modification) bases of single-stranded overhang; "Plasmid" = plasmid as positive control) in *E. coli* cell extract without GamS at 32 °C.

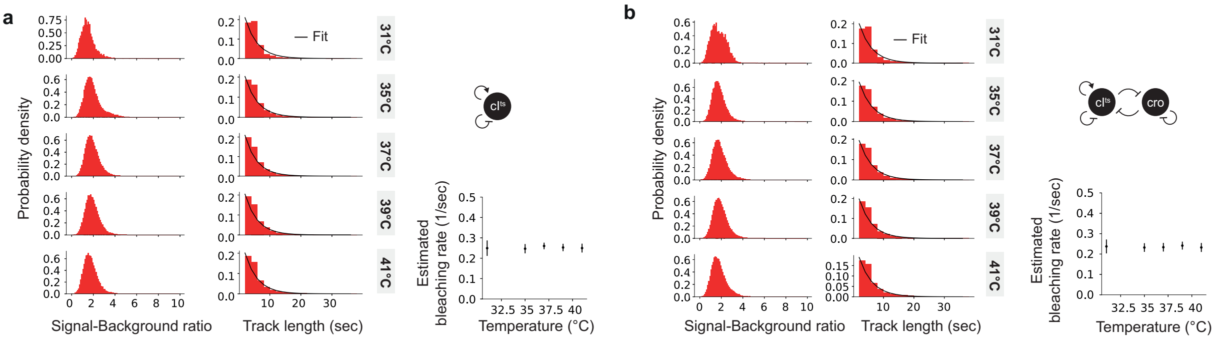

Supplementary Figure 4: Properties of individual cI^{ts}-mVenus proteins.

a, Signal-to-background ratios of single cI^{ts}-mVenus at the various temperatures with the monostable GRN (N=6702, 54388, 76421, 57164, 32177 for 31, 35, 37, 39, 41 °C, respectively). The time from the first encounter of a single mVenus spot till it vanished is computed for all spots, pooled and plotted as track length in the right column for the various temperatures (N=1608, 11148, 16438, 12180, 6310 for 31, 35, 37, 39, 41 °C, respectively). The track length distributions were fitted with a mono-exponential decay and gave values plotted against temperature in the lower right figure. Error bars show mean and SD as obtained from the fitting algorithm. **b**, Same analysis as in panel **a**, with signal-to-background ratio of single cI^{ts}-mVenus (N=7426, 26918, 57412, 126620, 42797) and track length (N=1329, 5739, 11494, 25826, 7770) of the bistable GRN with increasing temperature. Error bars for the bleaching rate estimation show mean and SD as obtained from the fitting algorithm.

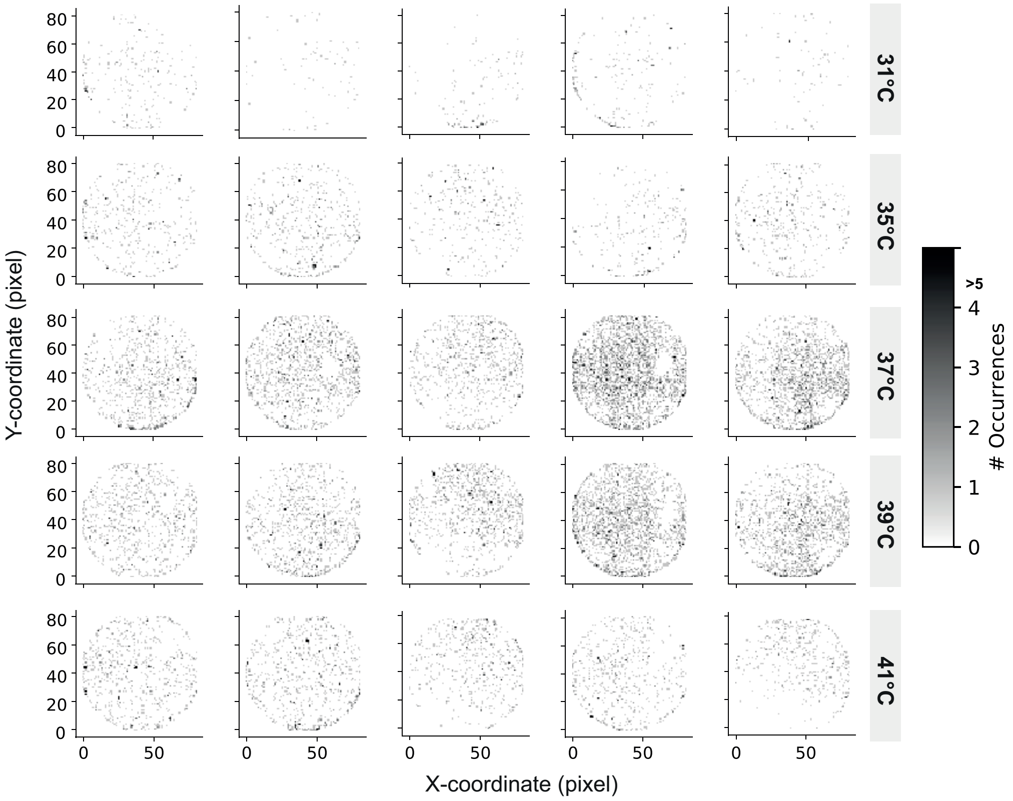

Supplementary Figure 5: Spatial distribution of single CI^{ts}-mVenus proteins.

2-D spatial histogram of spot counts integrated over the entire course of the experiment in 5 representative compartments at the indicated temperatures. Particles appeared evenly distributed at >37 °C due to unspecific adsorption of denatured and therefore sticky CI^{ts}-mVenus and non-optimal solution conditions. For other *in vitro* single-molecule studies, buffer and surface conditions can be readily changed towards minimizing unspecific adsorption(1), that was however not feasible due to the current working conditions of the cell-free extract.

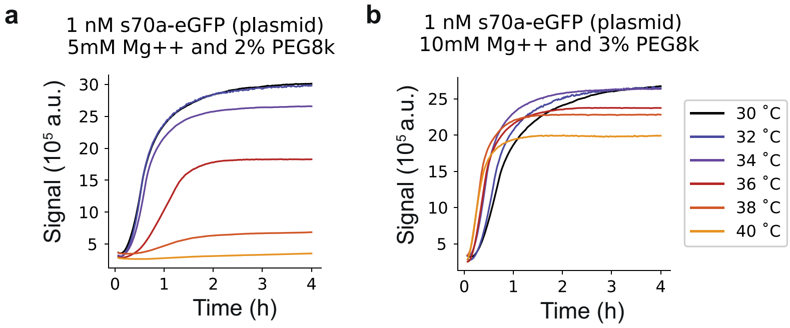

Supplementary Figure 6: Optimizing conditions for cell-free gene expression.

a, Fluorescent signal of GFP expression in solution experiments from plasmid at 1 nM concentration at various constant temperatures (color-coded) and 5 mM Mg^{2+} and 2% PEG8000 (crowding agent). **b**, Fluorescent signal of GFP expression in solution experiments from plasmid at 1 nM concentration at various constant temperatures (color-coded) and 10 mM Mg^{2+} and 3% PEG8000 (crowding agent). A higher concentration of Mg^{2+} and PEG8000 improved expression and led to similar amounts of GFP within the temperature range.

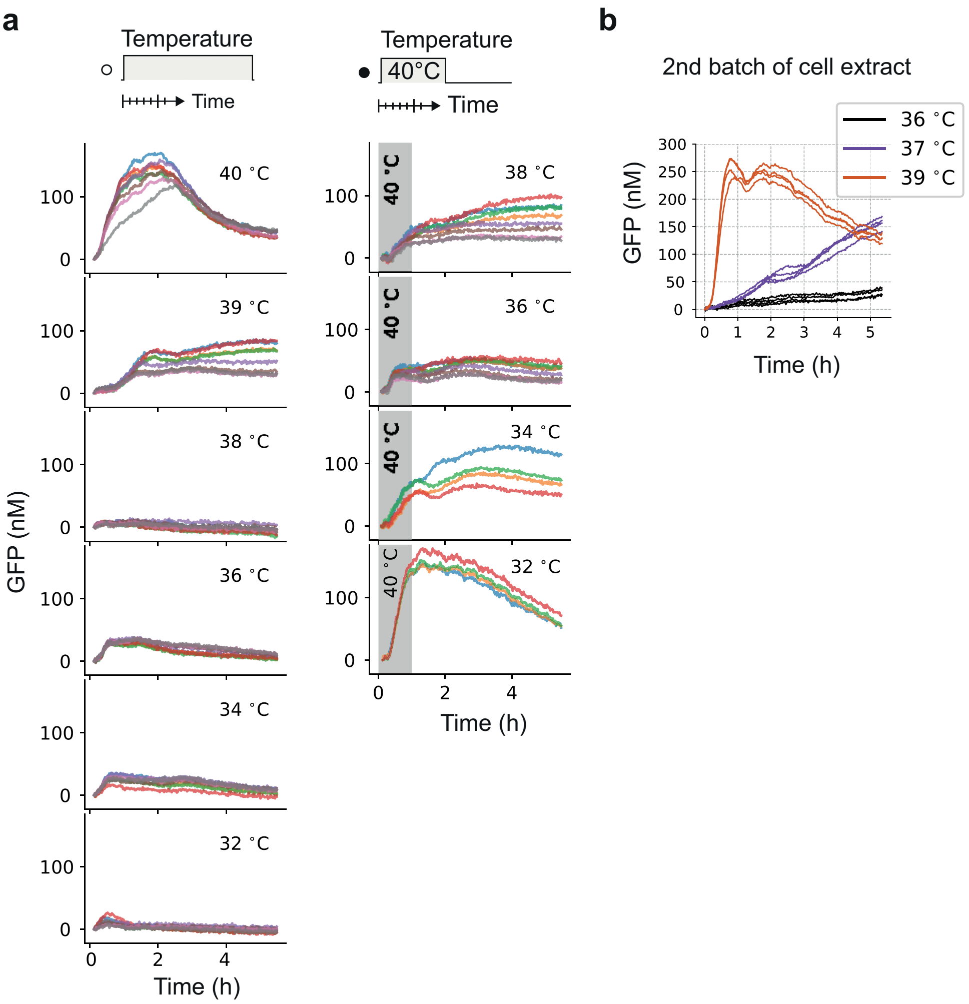

Supplementary Figure 7: Temperature response of the bistable GRN in the high-density regime.

a, The fluorescent signal of GFP under the P_R promoter in single compartments and various temperatures (first column). The bistability of the GRN was tested with initial protein expression at 40 °C (gray region in second column) and a drop in temperature (indicated on the right side in each plot of the second column). The slight variation in the initial response (<1h) is explained by the small variations of the microfluidic chips during the fabrication process. **b**, The precise GFP response was also obtained with a 2nd batch of cell-extract (third column).

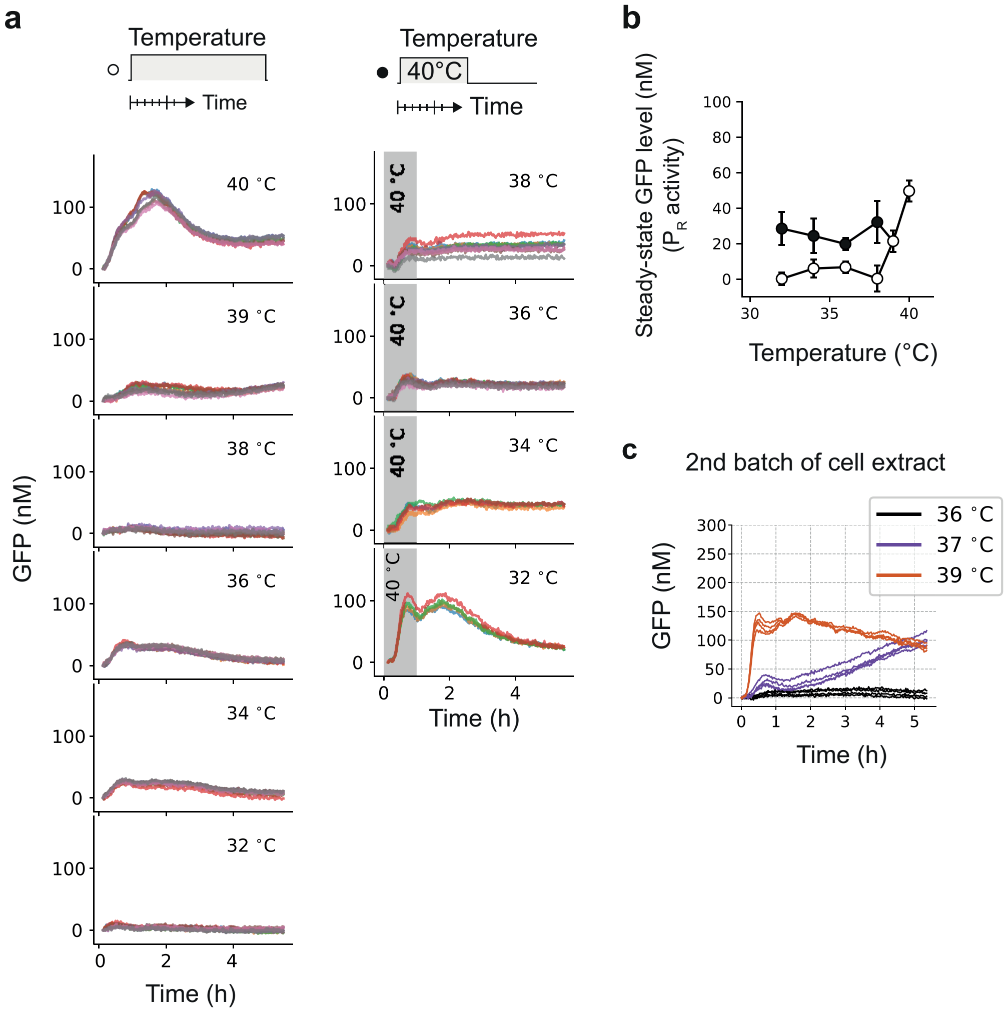

Supplementary Figure 8: Temperature response of the bistable GRN in the high-density regime at shorter compartment life-times.

a, The fluorescent signal of GFP under the P_R promoter in single compartments with 7 min protein life-time (see Supplementary Figure 1a) and various temperatures (first column). The bistability of the GRN was tested with initial protein expression at 40 °C (gray region in second column) and a drop in temperature (indicated on the right side in the subfigures of the second column). The slight variation in the initial response (<1h) is explained by the small variations of the microfluidic chips during the fabrication process. **b**, Steady-state values are plotted for the various temperature inputs with N=24. Error bars show mean and SD of compartments. **c**, The precise GFP response was also obtained with a 2nd batch of cell-extract.

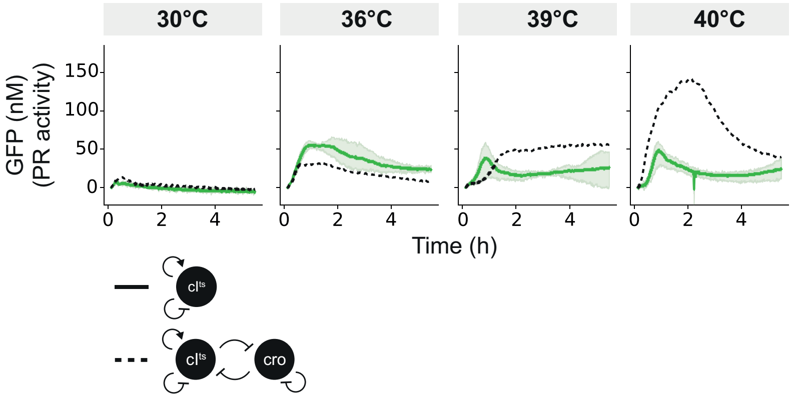

Supplementary Figure 9: Temperature response of the monostable GRN in the high-density regime.

The fluorescence signal of GFP under the P_R promoter without the *cro* gene in single compartments and various temperatures. The mean with minimum and maximum values for GFP dynamics of all compartments are shown as solid lines and shaded areas, respectively (N=24). The compartment mean GFP signals of the bistable GRN with the *cro* gene are shown as dashed line for comparison.

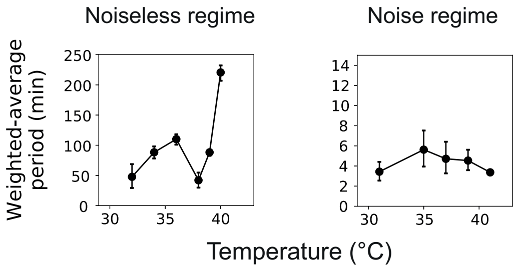

Supplementary Figure 10: Characteristic time scale of gene expression dynamics in the bistable GRN for the noiseless and noise regime.

A fast Fourier transform (FFT) of the full expression dynamics (with onset time) in the high gene density (left, N=24) and low gene density (right, for N see the bistable GRN in Supplementary Fig. 12) regimes was used to compute the frequency domain. The frequencies were then averaged with the weights of the corresponding FFT power spectrum (excluding the zero and noise frequencies, $0 < f_i < \text{min}^{-1}$) and plotted for all temperatures. Whereas the noiseless regime showed dynamics on a time scale of ~1 h, the dynamics in the noise regime happened on the ~5 min time scale. Error bars show mean and SD of compartments.

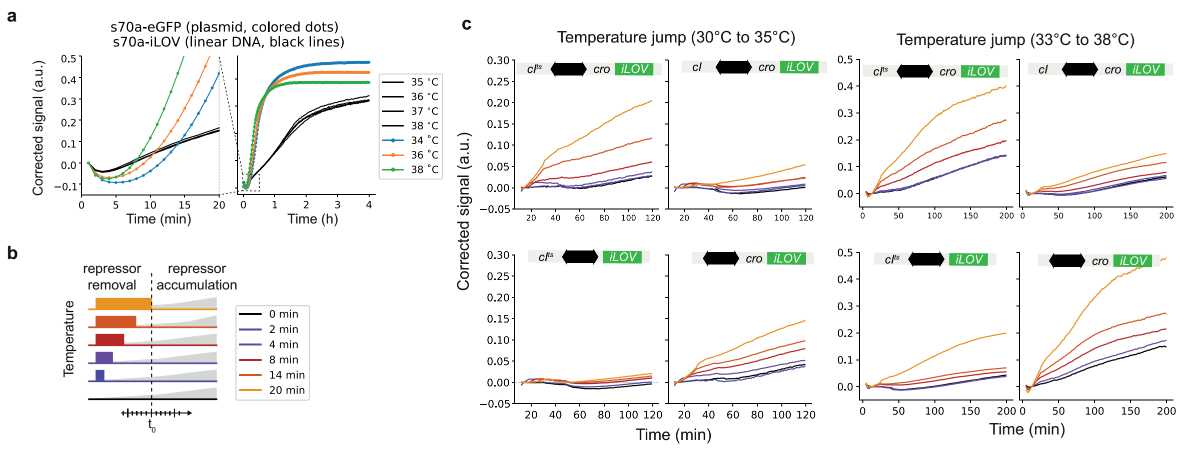

Supplementary Figure 11: Expression dynamics of the GRNs in solution experiments upon various temperature jumps.

a, The production dynamics of iLOV against the dynamics of GFP production in cell extract at various temperatures under a constitutive promoter. **b**, The experimental protocol to verify the dynamics of the GRNs in solution. The initial temperature jump deactivated temperature-sensitive CI^{ts} for various durations and the accumulation after the drop in temperature. **c**, Temperature jumps from 30/33 °C (left/right) to 35/38 °C (left/right) for the indicated GRNs (upper row: the bistable GRN with and without the temperature sensitive mutation, lower row: the monostable GRNs with the temperature-sensitive cI^{ts} or with cro). While the bistable wild-type GRN showed little temperature dependence compared to the bistable GRN due to the missing temperature mutation, the monostable GRN without the *cro* gene is not dependent on temperature since the GRN can return to their initial promoter activity.

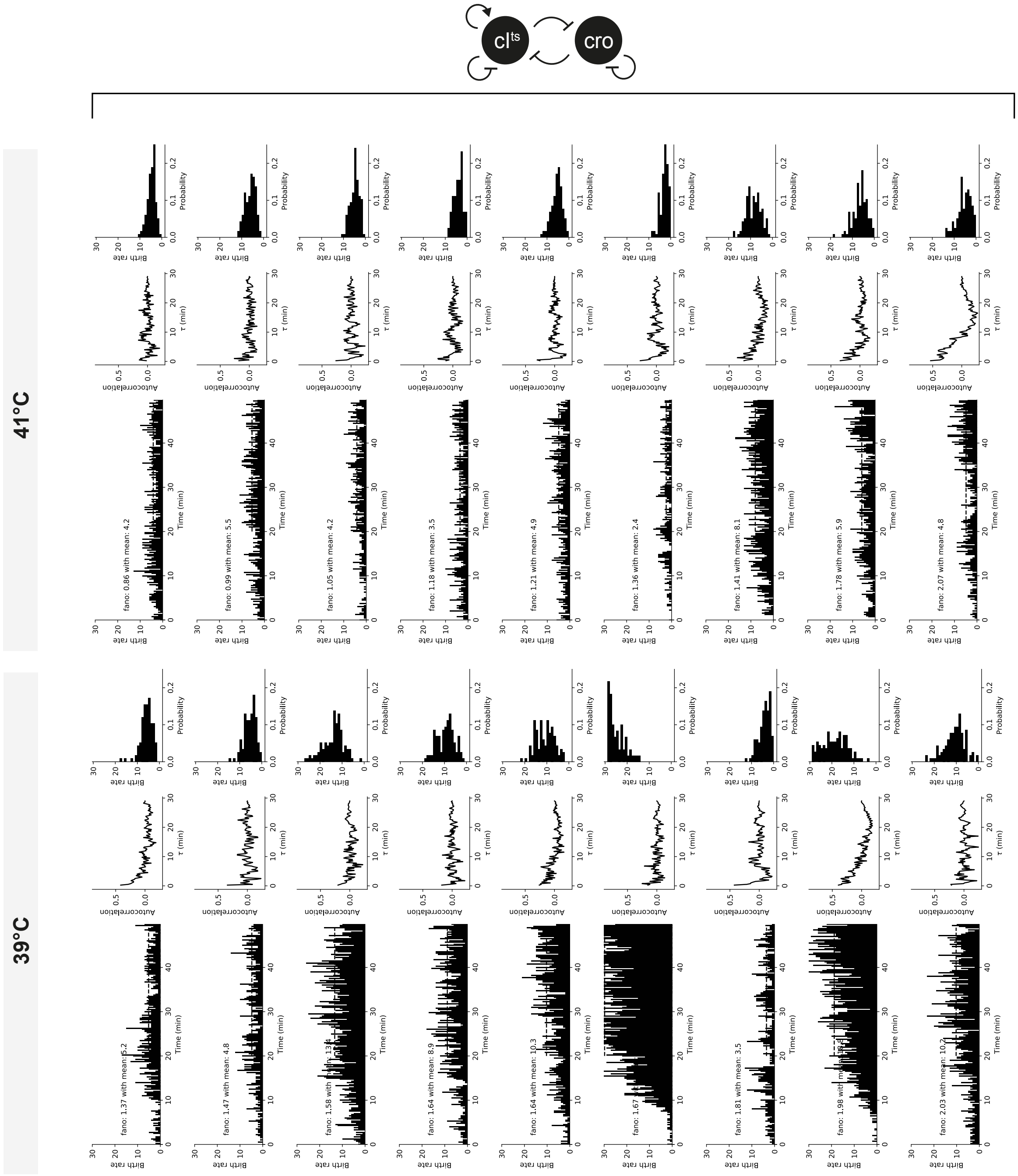
cI^ts
cro
41°C
39°C

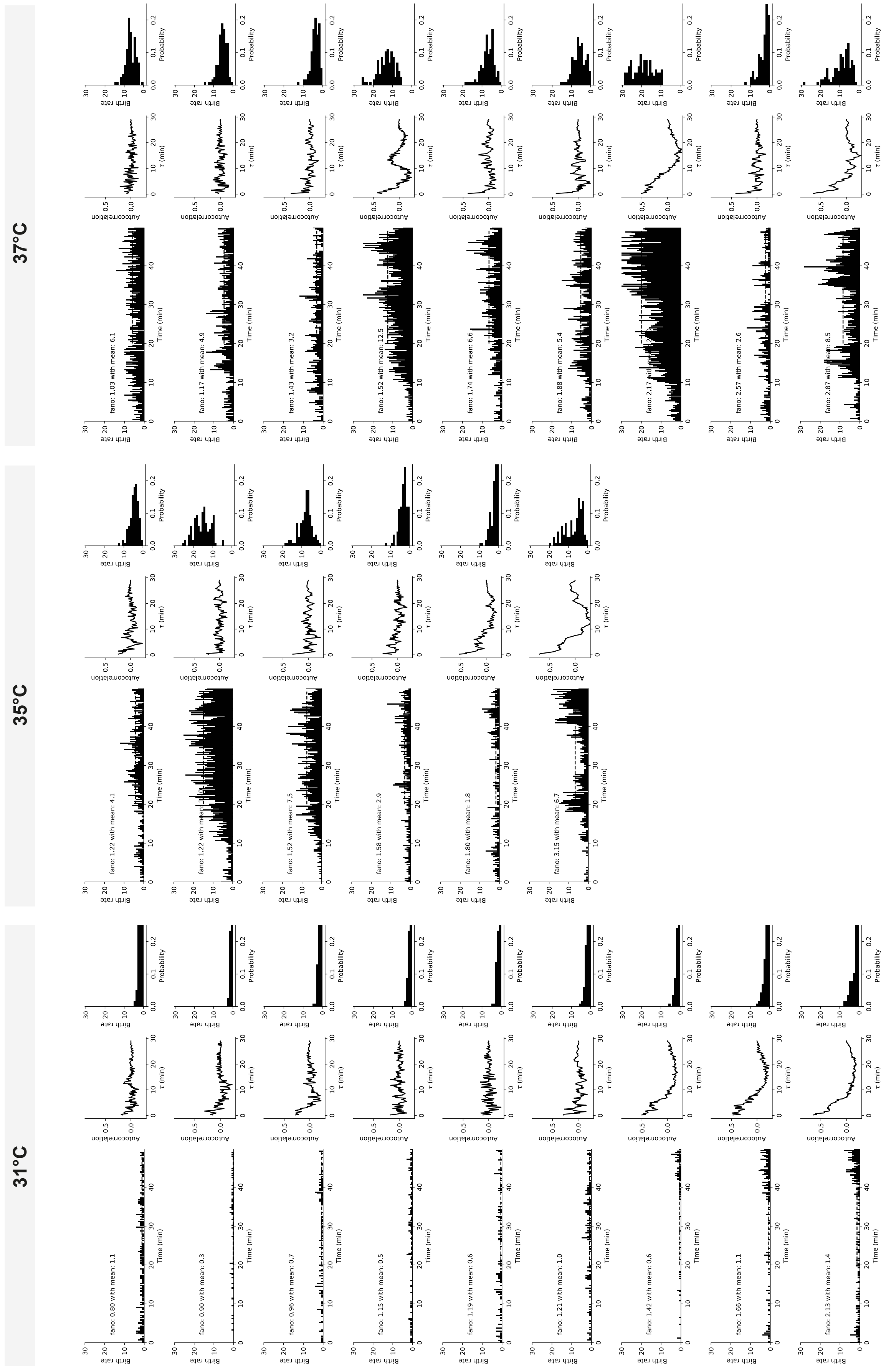
31°C
35°C
37°C
fano: 0.80 with mean: 1.1
fano: 0.90 with mean: 0.3
fano: 0.96 with mean: 0.7
fano: 1.15 with mean: 0.5
fano: 1.19 with mean: 0.6
fano: 1.21 with mean: 1.0
fano: 1.42 with mean: 0.6
fano: 1.66 with mean: 1.1
fano: 2.13 with mean: 1.4
fano: 1.22 with mean: 4.1
fano: 1.22 with mea
fano: 1.52 with mean: 7.5
fano: 1.58 with mean: 2.9
fano: 1.80 with mean: 1.8
fano: 3.15 with mean: 6.7
fano: 1.03 with mean: 6.1
fano: 1.17 with mean: 4.9
fano: 1.43 with mean: 3.2
fano: 1.52 with mean: 12.5
fano: 1.74 with mean: 6.6
fano: 1.88 with mean: 5.4
fano: 2.17
fano: 2.57 with mean: 2.6
fano: 2.87 with mean: 8.5
Birth rate
Time (min)
Autocorrelation
τ (min)
Probability

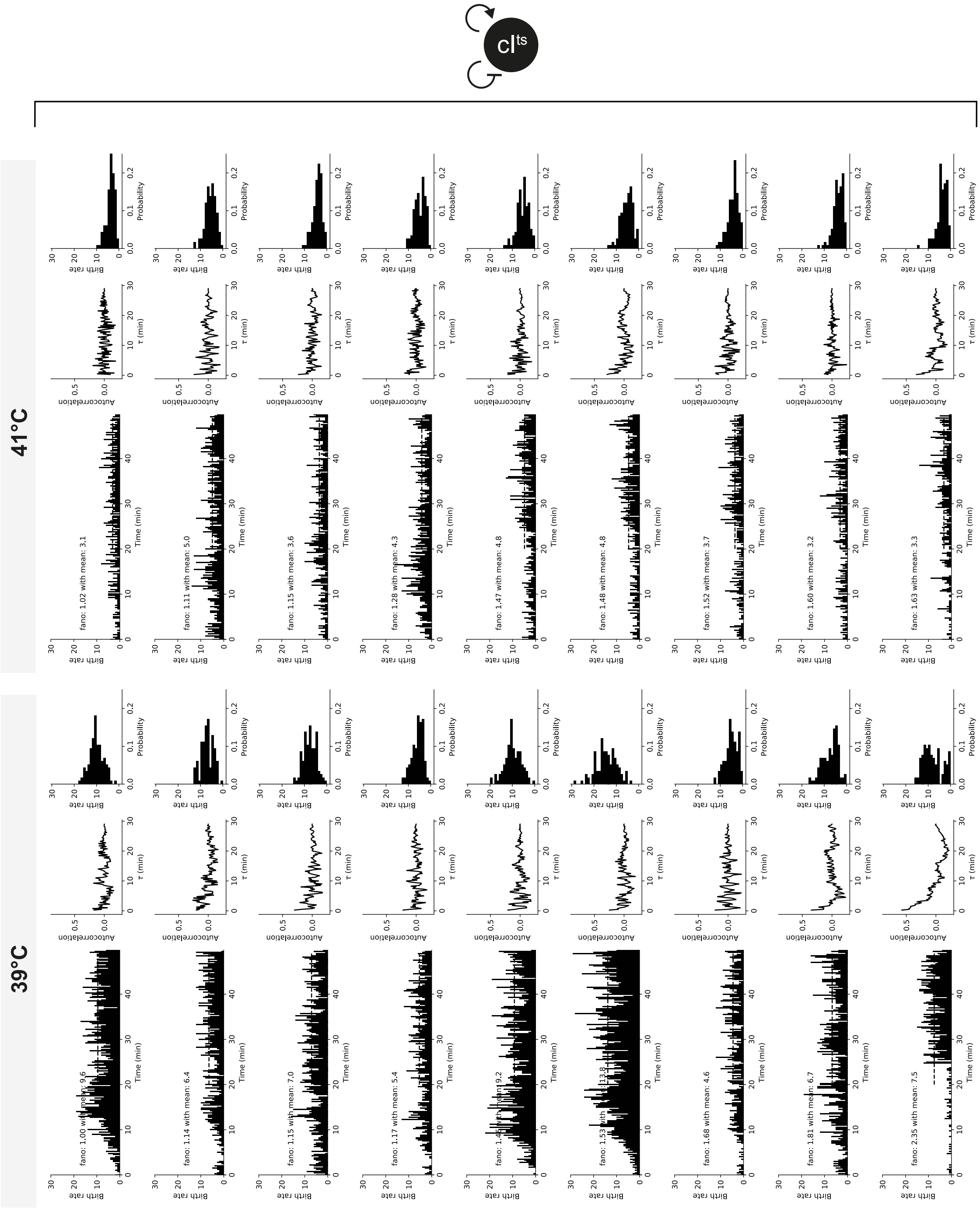

cl^ts
39°C
41°C
fano: 1.14 with mean: 6.4
fano: 1.17 with mean: 5.4
fano: 1.68 with mean: 4.6
fano: 2.35 with mean: 7.5
fano: 1.02 with mean: 3.1
fano: 1.11 with mean: 5.0
fano: 1.15 with mean: 3.6
fano: 1.28 with mean: 4.3
fano: 1.47 with mean: 4.8
fano: 1.48 with mean: 4.8
fano: 1.52 with mean: 3.7
fano: 1.60 with mean: 3.2
fano: 1.63 with mean: 3.3
Birth rate
Time (min)
Autocorrelation
τ (min)
Probability

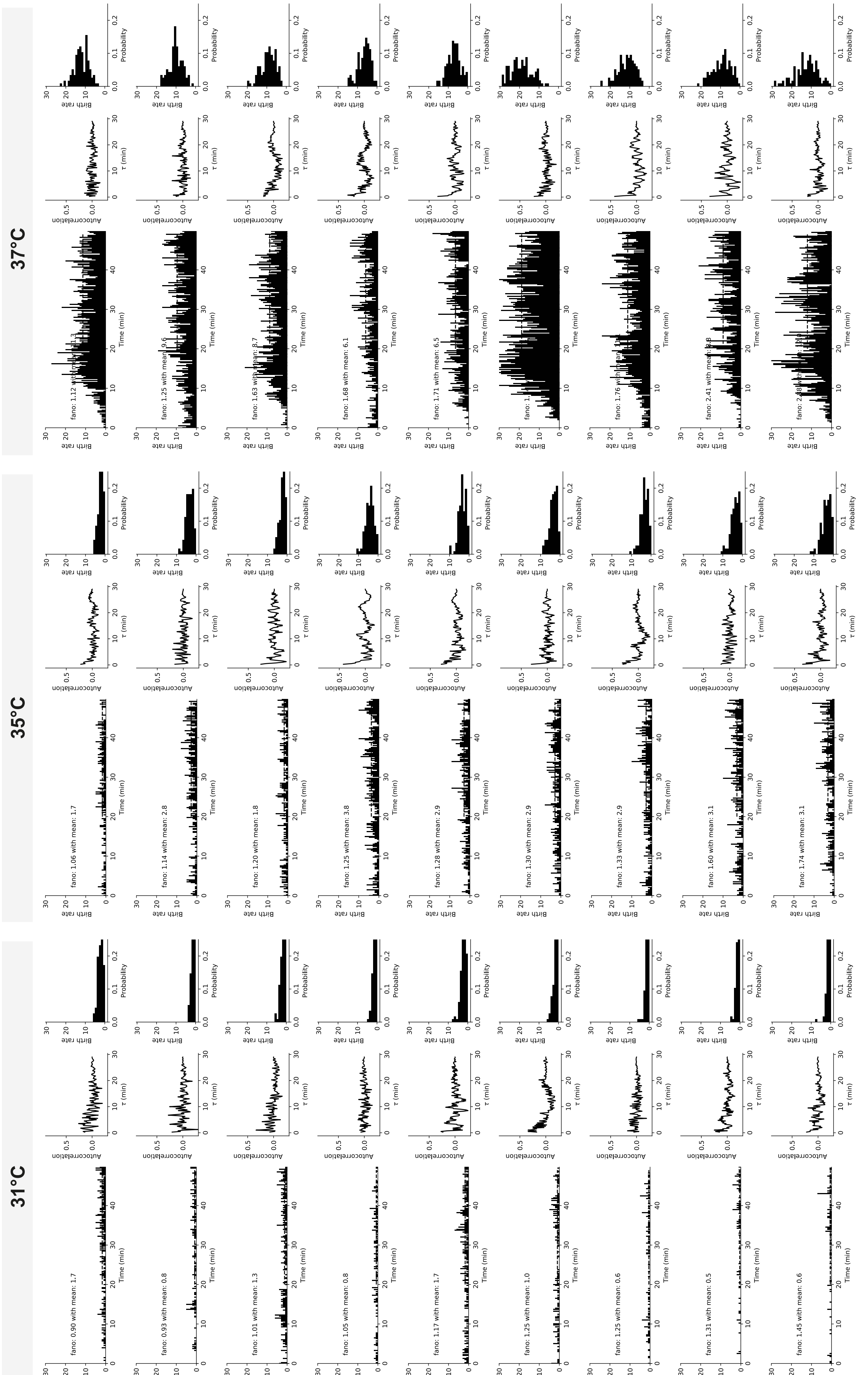
31°C
35°C
37°C
Birth rate
Time (min)
Autocorrelation
τ (min)
Probability
fano: 0.90 with mean: 1.7
fano: 0.93 with mean: 0.8
fano: 1.01 with mean: 1.3
fano: 1.05 with mean: 0.8
fano: 1.17 with mean: 1.7
fano: 1.25 with mean: 1.0
fano: 1.25 with mean: 0.6
fano: 1.31 with mean: 0.5
fano: 1.45 with mean: 0.6
fano: 1.06 with mean: 1.7
fano: 1.14 with mean: 2.8
fano: 1.20 with mean: 1.8
fano: 1.25 with mean: 3.8
fano: 1.28 with mean: 2.9
fano: 1.30 with mean: 2.9
fano: 1.33 with mean: 2.9
fano: 1.60 with mean: 3.1
fano: 1.74 with mean: 3.1
fano: 1.68 with mean: 6.1
fano: 1.71 with mean: 6.5

Supplementary Figure 12: Examples of single compartments with production rates at different temperatures and the bistable and monostable GRNs.

The time-averaged production rates (shown in subfigures), Fano factor (shown in subfigures), ACFs and probability distributions are computed from the region as indicated by the dashed line. Total number of experiments for the bistable (upper two pages) and monostable (lower two pages) GRN in the order from 31 to 41 °C were 9, 6, 9, 18, 9 and 9, 12, 9, 9, 9, respectively.

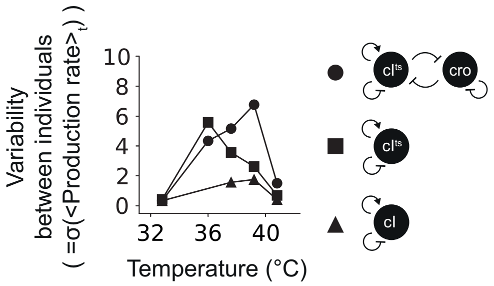

Supplementary Figure 13: Variability between individual cell models.

The variability was computed as standard deviation from the time-averaged production rates of individual compartments for the various temperatures and indicated GRNs.

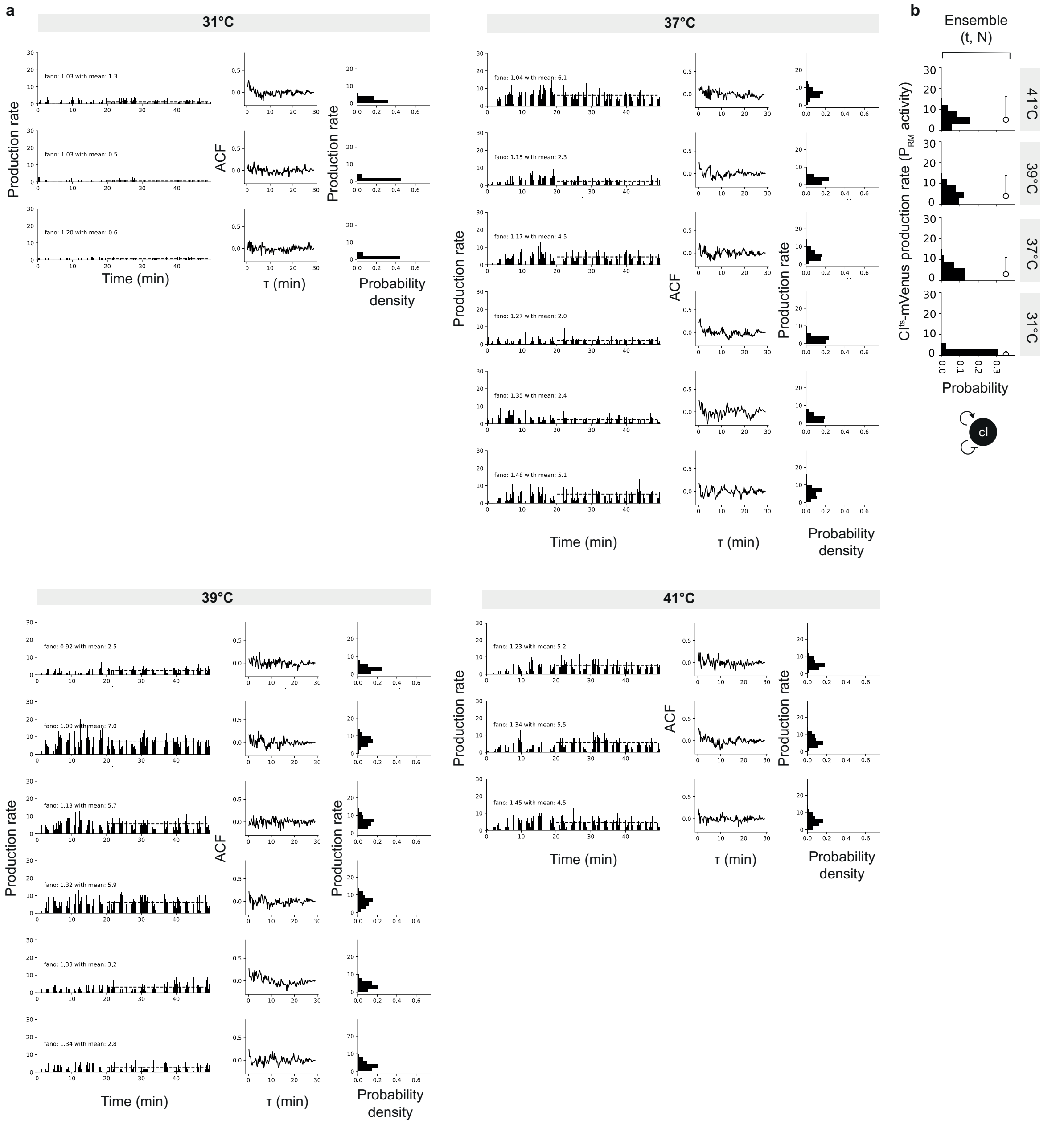

Supplementary Figure 14: Single compartments with production rates at different temperatures with the wild-type monostable GRN.

a, Production rates, ACF, and histogram of production rates (from left to right) for individual cell models at various temperatures. The time-averaged production rates, Fano factor (shown in subfigures), ACFs and probability distributions are computed during the time window as indicated by the dashed line. **b**, The ensemble production rate of the wild-type monostable GRN as in Fig. 2c and Fig. 3a in the main text. Total number of experiments in the order from 31 to 41 °C were 3, 6, 6, 3. Circle with error bars give the median and the 32th to 68th percentile of the ensemble production rates (N=348, 696, 696, 348 in the order of increasing temperature).

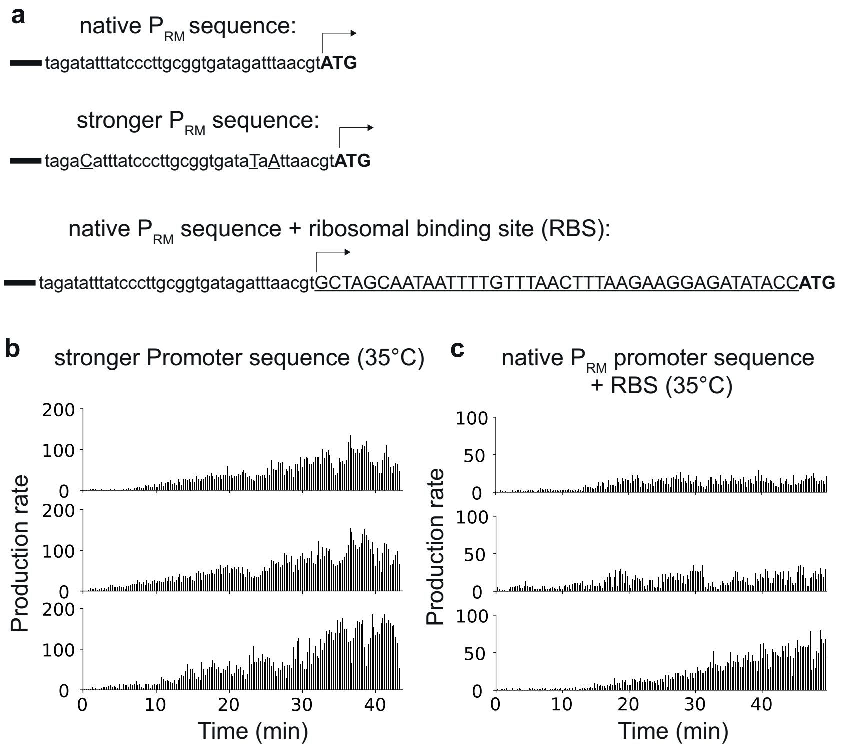

Supplementary Figure 15: Production rates in single compartments with change of transcription and translation elements in the monostable GRN without the *cro* gene.

a, Wild-type sequence of P_{RM}, a consensus promoter for σ_{70}, and the native promoter with ribosomal binding site at the 5'-UTR of cI^{ts}. Transcription start site is highlighted by the arrow. **b**, Proteins produced with the consensus promoter sequence. The signal was measured as in Fig. 1e, Fig. 2c, Fig. 3a, and Fig. 4c. **c**, Proteins produced with the RBS and native P_{RM}. The signal was measured as in Fig. 1e, Fig. 2c, Fig. 3a, and Fig. 4c.

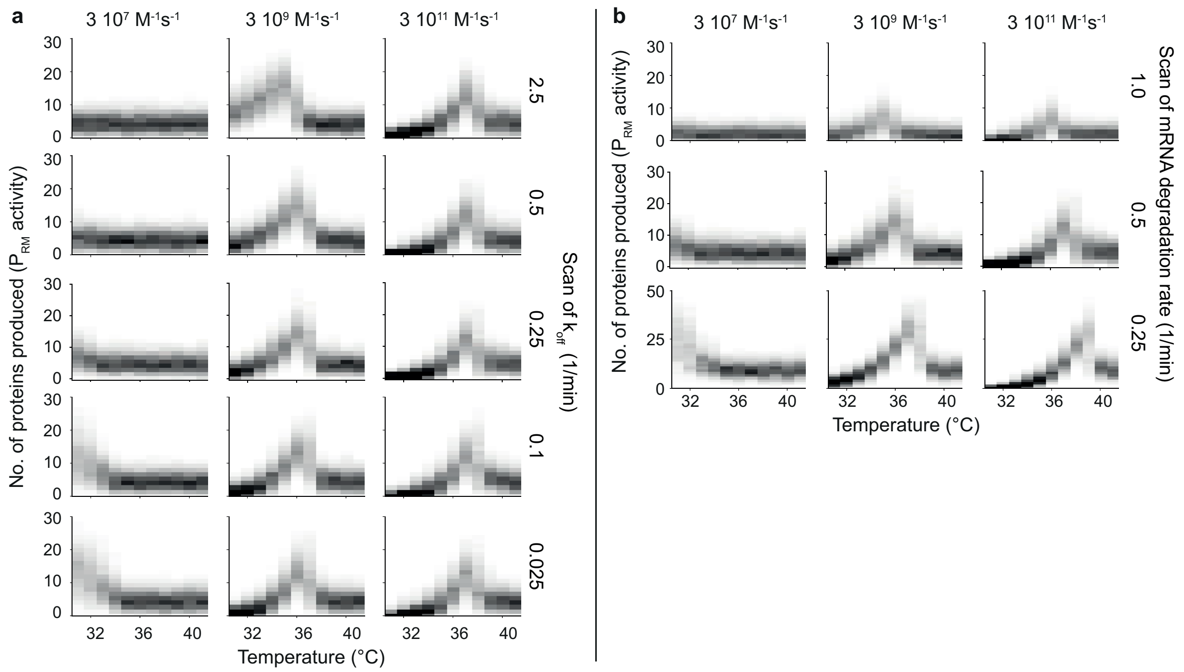

Supplementary Figure 16: Stochastic simulation of the monostable GRN with various parameter scans.

a, Scan of k_{off} rates of CI (0.25 min^{-1} was used in the main text as described in the methods part and the supplementary information). The simulated shape of the CI self-regulation showed no major effect from a scan in k_{off} rate. **b**, Scan of mRNA degradation rates (0.5 min^{-1} was used in the main text as described in the methods part and the supplementary information). The simulated shape of the CI self-regulation showed no major effect from a scan in mRNA degradation rate. The amplitude of protein production rates increased as expected with longer mRNA half-life.

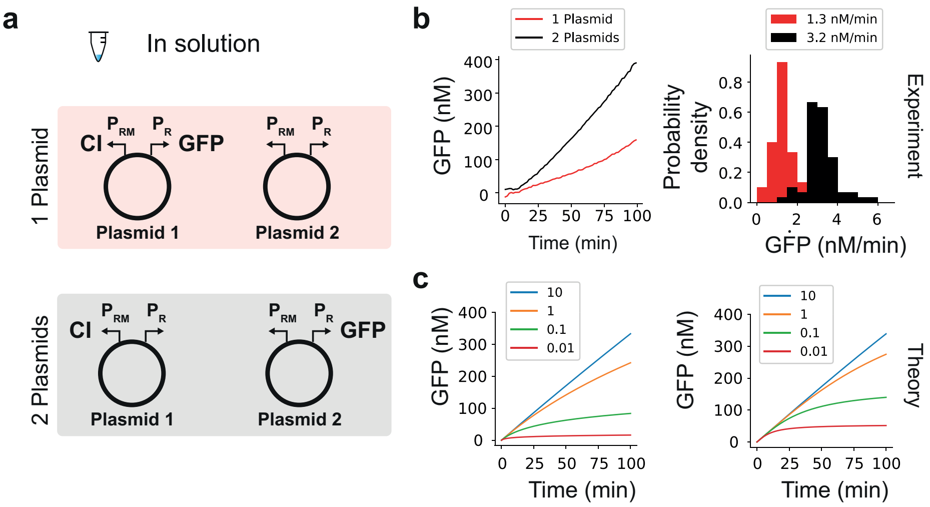

Supplementary Figure 17: Solution experiment with co-expression of CI^{ts} and GFP on same and different plasmids.

a, First set (red region): The first plasmid encoded both CI and GFP with a promoter architecture as in the bistable GRN. The other plasmid encoded no gene, but leveled the total DNA concentration. Second set (black region): The first plasmid only encoded CI under the native P_{RM} promoter architecture. The other plasmid encoded GFP under the P_R promoter with the native promoter architecture. **b**, Protein expression was performed in cell extract at 32 °C (no deactivation) with two different pairs of plasmids. Increase of GFP signal over time is shown (first plasmid set = red, second plasmid set = black). The data was clipped to the linear expression regime (the dynamics of closed systems eventually slow down due to energy consumption). The rate of GFP production is plotted with the average rates indicated in the figure legend (right plot). **c**, Theoretical dynamics of equilibrium binding of CI to DNA (Methods). Comparing the experimental data to different thermodynamic equilibrium models without (n=1, left plot) and with (n=2, right plot) cooperativity gave an estimate of ~100-fold lower K_D value (values in legend).

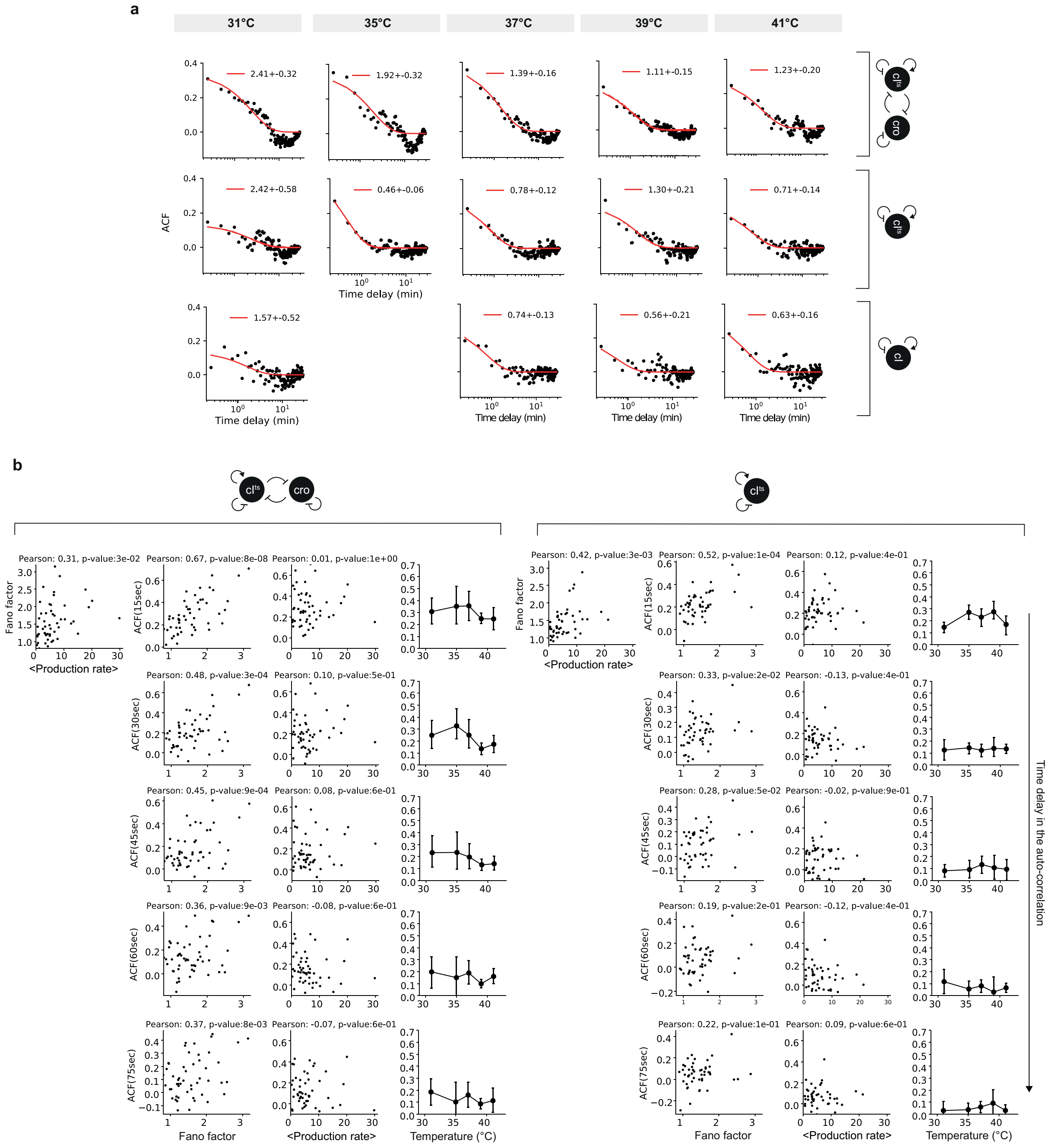

Supplementary Figure 18: Ensemble-averaged autocorrelation function, correlations of time-averaged production rates, and Fano factor, and auto-correlation amplitudes at different time delays for the different GRNs.

a, Ensemble-averaged ACFs from Supplementary Figure 12 at various temperatures and fits to a mono-exponential decay (red line) for the three indicated GRNs (estimated decay constant in minutes as show in figure legends). **b**, Left (bistable GRN) and right (monostable GRN) column show Fano factor against time-averaged production rates, ACF amplitude at the selected time delay against Fano factor, ACF amplitude at the selected time delay against time-averaged production rates, and ACF amplitude at the

selected time delay against temperature (from left to right). Pearson correlation and p values are given in the title of each plot. The time delay is given in the y label in each GRN column (15, 30, 45, 60, 75 sec from top to bottom). Error bars were bootstrapped and show mean and SD of compartments (for N see the bistable and monostable GRN in Supplementary Fig. 12).

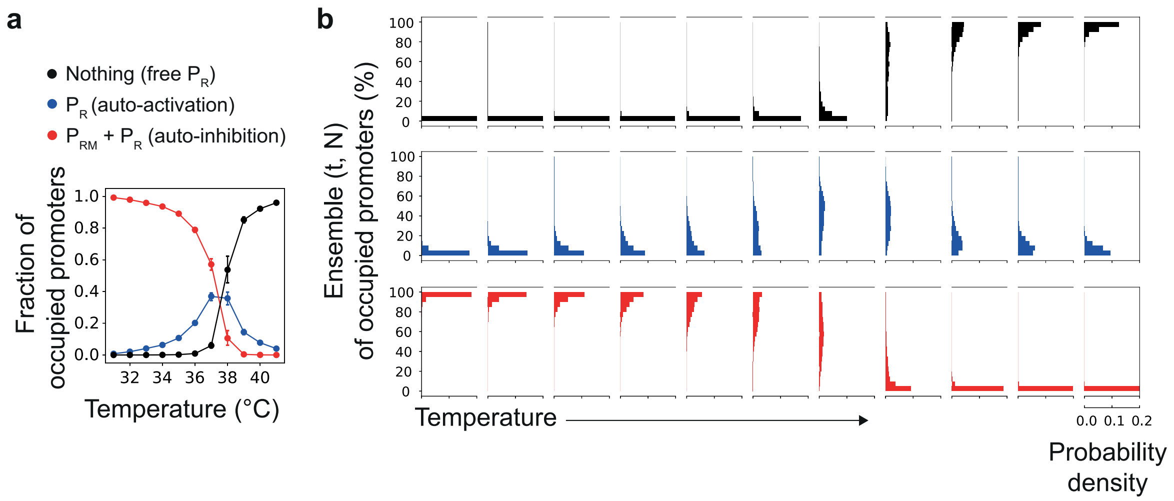

Supplementary Figure 19: Simulated promoter occupancies with temperature.

a, Averaged fraction of occupied promoters (time-averaged) by CI as obtained from simulation (as in Fig. 3D and Supplementary Table 3 with k_{ON}=3e-15) for three different categories: No CI is bound to P_R (black line), CI is bound to the P_R promoter (blue line), and CI is bound to P_R and P_{RM} (red line). Errorbars were bootstrapped and indicate mean and SD of simulated compartments (N=20). **b**, Simulated ensemble promoter occupancy as sampled over time t and compartment N at various temperatures (left to right: 1 °C steps from 31 °C to 41 °C). The occupancies are given for free P_R (upper row), bound P_R (center row), and bound P_R and P_{RM} (lower row). A transiently unoccupied (free) P_R promoter would allow leaky production of Cro already at ~35 °C.

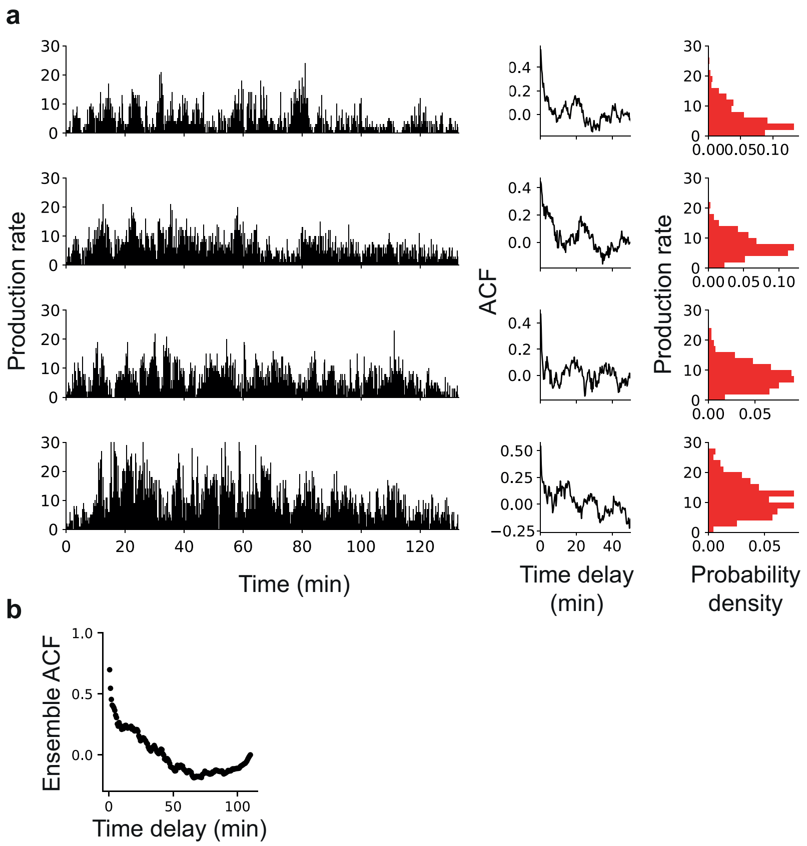

Supplementary Figure 20: Long-term expression dynamics of the bistable GRN at 37 °C.
a, Individual production rates, individual ACFs, individual probability distributions of production rates (left to right) **b,** Ensemble-averaged ACF of all compartments. The negative auto-correlation at 50-100 min may stem from slow degradation of DNA, also indicated by the slow decay of production rates in the individual production rates.

Supplementary Table 1: Primer sequences.

Name	Sequence	Mod	Usage
P1	GGAGATGGCGCCCAACAGTCGCAATTCAGAGCGGCAGCAAGTG	Internal Modification	Circular DNA
pP1	GTGGATAACCGTATTACCGCCTTTGAGTG	5'-Pi	Circular DNA
P2	GACTGTTGGGCGCCATCTCCGTGGATAACCGTATTACCGCCTTTGAGTG	Internal Modification	Circular DNA
pP2	GCAATTCAGAGCGGCAGCAAGTG	5'-Pi	Circular DNA
P3	CGCCGCAGAGTGGATGTACAGAAAAGCCCGCCTTTCGGCGGGCTTTGCTCGAGTTATCAGCCAAACGTCTCTTCAGGC		Lift-off from lambda genome
P4	TATATCTCCTTCTTAAAGTTAAACAAAATTATTGCTAGCTTATGCTGTTGTTTTTTTGTTACTCGGGAAG		Lift-off from lambda genome

Supplementary Table 2: Ensemble averaged production rates for the various GRNs. The values are given in the following order: 68th / *median* / 32th.

Temperature *(℃)*	Bistable GRN	Monostable GRN	Wild-type monostable GRN
41	6 / 4 / 3	5 / 4 / 3	6 / 5 / 4
39	10 / 7 / 4	10 / 7 / 5	6 / 4 / 3
37	9 / 6 / 4	12 / 9 / 7	5 / 3 / 2
35	8 / 5 / 3	5 / 3 / 2	-
31	1 / 0 / 0	1 / 1 / 0	1 / 0.5 / 0

Supplementary Table 3: Parameters for stochastic simulations.

Parameters	*Values*	*Units*	*Reference*
Basal transcription rate	0.09	min^{-1}	Estimated by the time-averaged production rate at 41 °C in the monostable GRN
Auto-activated transcription rate	0.9	min^{-1}	Estimated by the time-averaged production rate at 37 °C in the monostable GRN
k_{on} for O_R2 and O_R1	3e-11 to 3e-15	l/(#*min)	Lowest value taken from (2)
k_{on} for O_R3	2e-11 to 2e-15	l/(#*min)	Estimated from (2) and to integrate the fact that CI has a lower affinity to O_R3 than O_R2 and O_R1 (3)
k_{off}	0.25	min^{-1}	~1.5 1/min (2)
Translation rate	1.3	min^{-1}	~3 proteins per mRNA was estimated and supported by (4) with 6 proteins per mRNA considering lower expression activity in cell-free extract
mRNA degradation rate	0.5	min^{-1}	(5)
Amount of DNA	20	#	This work
Protein life-time and deactivation rate	0.04*exp(0.55*(T-32))	min^{-1}	(6); We assumed a longer protein life-time (25 against ~10 min) due to hindered diffusion from DNA binding.
Compartment volume	3.8e-12	l	This work

REFERENCES

1. Hua, B. *et al.* An improved surface passivation method for single-molecule studies. *Nat. Methods* **11,** 1233–1236 (2014).
2. Nelson, H. C. M. & Sauer, R. T. Lambda repressor mutations that increase the affinity and specificity of operator binding. *Cell* **42,** 549–558 (1985).
3. Ptashne, M. *A genetic switch 3rd edition*. *CSHL Press* (2004). doi:10.1038/nrc1424
4. Sepúlveda, L. A., Xu, H., Zhang, J., Wang, M. & Golding, I. Measurement of gene regulation in individual cells reveals rapid switching between promoter states. *Science* **351,** 1218–1222 (2016).
5. Karzbrun, E., Shin, J., Bar-Ziv, R. H. & Noireaux, V. Coarse-grained dynamics of protein synthesis in a cell-free system. *Phys. Rev. Lett.* **106,** 48104 (2011).
6. Isaacs, F. J., Hasty, J., Cantor, C. R. & Collins, J. J. Prediction and measurement of an autoregulatory genetic module. *Proc. Natl. Acad. Sci. U. S. A.* **100,** 7714–7719 (2003).
