# Supplementary material for: From deterministic to fuzzy decision-making in artificial cells
Source: Nat Commun. 2020 Nov 6;11:5648. doi: 10.1038/s41467-020-19395-4 (PMC7648101; doi:10.1038/s41467-020-19395-4)
Supplement: Supplementary file 2 — Reporting Summary [file 41467_2020_19395_MOESM2_ESM.pdf]

nature research

Corresponding author(s): Roy Bar-Ziv

Last updated by author(s): Sep 30, 2020

Reporting Summary

Nature Research wishes to improve the reproducibility of the work that we publish. This form provides structure for consistency and transparency in reporting. For further information on Nature Research policies, see our Editorial Policies and the Editorial Policy Checklist.

Statistics

For all statistical analyses, confirm that the following items are present in the figure legend, table legend, main text, or Methods section.

n/a	Confirmed	
[ ]	[x]	The exact sample size (n) for each experimental group/condition, given as a discrete number and unit of measurement
[ ]	[x]	A statement on whether measurements were taken from distinct samples or whether the same sample was measured repeatedly
[x]	[ ]	The statistical test(s) used AND whether they are one- or two-sided<br>*Only common tests should be described solely by name; describe more complex techniques in the Methods section.*
[x]	[ ]	A description of all covariates tested
[x]	[ ]	A description of any assumptions or corrections, such as tests of normality and adjustment for multiple comparisons
[ ]	[x]	A full description of the statistical parameters including central tendency (e.g. means) or other basic estimates (e.g. regression coefficient) AND variation (e.g. standard deviation) or associated estimates of uncertainty (e.g. confidence intervals)
[x]	[ ]	For null hypothesis testing, the test statistic (e.g. F, t, r) with confidence intervals, effect sizes, degrees of freedom and P value noted<br>*Give P values as exact values whenever suitable.*
[x]	[ ]	For Bayesian analysis, information on the choice of priors and Markov chain Monte Carlo settings
[x]	[ ]	For hierarchical and complex designs, identification of the appropriate level for tests and full reporting of outcomes
[x]	[ ]	Estimates of effect sizes (e.g. Cohen's d, Pearson's r), indicating how they were calculated

Our web collection on statistics for biologists contains articles on many of the points above.

Software and code

Policy information about availability of computer code

Data collection	We used Micro-Manager 1.4 and Python v3.7 (with PyQt5) for automated DNA patterning in the high DNA density regime. We used Zen (Carl Zeiss, Germany) and Andor Solis (Oxford Instruments) for image acquisition in the high and low DNA density regime, respectively. We used an Arduino control software (on Github, Nicovich, P. R., Walsh, J., Böcking, T. & Gaus, K. NicoLase - An open-source diode laser combiner, fiber launch, and sequencing controller for fluorescence microscopy. PLoS One 12, 1–15 (2017)) for synchronization of lasers and camera exposure. We used StepOne and StepOnePlus v2.3 (Applied Biosystems) for the RT-PCR (temperature jumps) experiments and ClarioStar Plus (BMG Labtech) for the closed system experiments. We used COPASI 4.20 for the stochastic stimulations.
Data analysis	We used an adapted version of Picasso (on Github, Schnitzbauer, J., Strauss, M. T., Schlichthaerle, T., Schueder, F. & Jungmann, R. Super-resolution microscopy with DNA-PAINT. Nat. Protoc. 12, 1198–1228 (2017)) for single-molecule localization, the Fiji distribution of ImageJ 1.51p for image analysis in the high DNA density regime, and Python v3.7 (with Seaborn v0.9, Matplotlib v3.3, NumPy v1.16, Scipy v1.3, OpenCV v4.4, and scikit-image v0.15) for all data analysis.

For manuscripts utilizing custom algorithms or software that are central to the research but not yet described in published literature, software must be made available to editors and reviewers. We strongly encourage code deposition in a community repository (e.g. GitHub). See the Nature Research guidelines for submitting code & software for further information.

Data

Policy information about availability of data

All manuscripts must include a data availability statement. This statement should provide the following information, where applicable:

- Accession codes, unique identifiers, or web links for publicly available datasets
- A list of figures that have associated raw data
- A description of any restrictions on data availability

The authors declare that the data supporting the findings of this study are available within the paper and its supplementary information files. Raw images are available from the corresponding author upon reasonable request. Source data are provided with this paper.

Field-specific reporting

Please select the one below that is the best fit for your research. If you are not sure, read the appropriate sections before making your selection.

[x] Life sciences [ ] Behavioural & social sciences [ ] Ecological, evolutionary & environmental sciences

For a reference copy of the document with all sections, see nature.com/documents/nr-reporting-summary-flat.pdf

Life sciences study design

All studies must disclose on these points even when the disclosure is negative.

Sample size	No statistical methods were used to calculate sample size. Typical experiments were repeated at least three times with 3 isolated (independent) compartments in each microfluidic chip (>9 times for each condition as indicated in the supplementary information), with the exception of partial data presented in Fig. S6, Fig. S11, Fig. S14, and Fig. S16. The experimental distributions converged and the number of replicates is on par with standard biophysical experiments in the field.
Data exclusions	No data was excluded from analysis.
Replication	A minimum of six replicates for main text figures were used and are indicated in the figure legends. Experiments were reliably reproduced over an extended period of time with different microfluidic chips, preparations of the cell-free expression system, and DNA assemblies.
Randomization	Not applicable to this study because group allocation was not carried out. Temperatures and genetic circuit variants were randomized across lanes in each microfluidic chip.
Blinding	Blinding was not applied in this proof-of-concept study. Not applicable to this study because group allocation was not carried out.

Reporting for specific materials, systems and methods

We require information from authors about some types of materials, experimental systems and methods used in many studies. Here, indicate whether each material, system or method listed is relevant to your study. If you are not sure if a list item applies to your research, read the appropriate section before selecting a response.

Materials & experimental systems

n/a	Involved in the study	
[x]	[ ]	Antibodies
[x]	[ ]	Eukaryotic cell lines
[x]	[ ]	Palaeontology and archaeology
[x]	[ ]	Animals and other organisms
[x]	[ ]	Human research participants
[x]	[ ]	Clinical data
[x]	[ ]	Dual use research of concern

Methods

n/a	Involved in the study	
[x]	[ ]	ChIP-seq
[x]	[ ]	Flow cytometry
[x]	[ ]	MRI-based neuroimaging
