# Supplementary figures and images for: From deterministic to fuzzy decision-making in artificial cells
Source: Nat Commun. 2020 Nov 6;11:5648. doi: 10.1038/s41467-020-19395-4 (PMC7648101; doi:10.1038/s41467-020-19395-4)

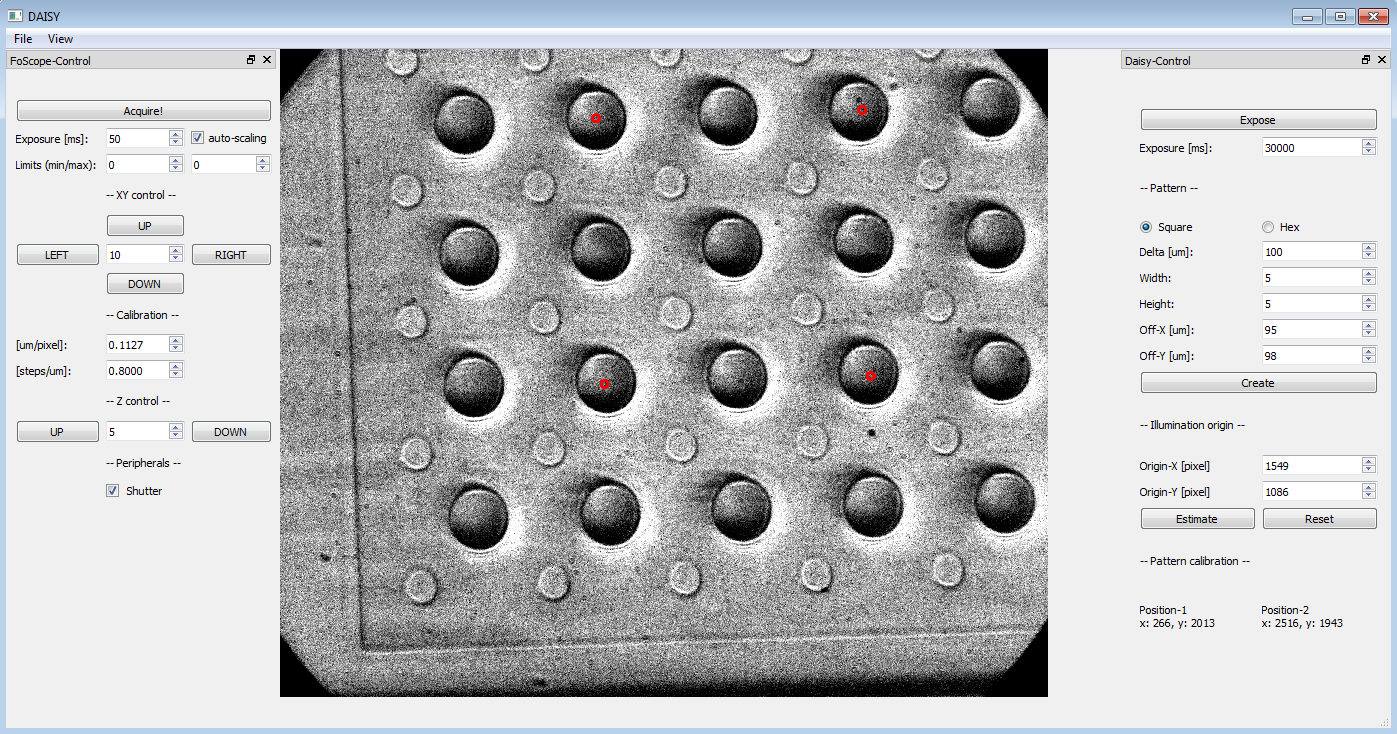

Supplement: Supplementary file 4 — Source Data [file 41467_2020_19395_MOESM4_ESM.zip › source-data/uManager-lithography/gui-screenshot.png]
